# Supplementary material for: iNaturalist mammal observations classified by evidence type
Source: PLoS One. 2026 Jul 29;21(7):e0353282. doi: 10.1371/journal.pone.0353282 (PMC13419177; doi:10.1371/journal.pone.0353282)
Supplement: Peer review history

# Author comment: Author response to Decision Letter 0

**DOI:** 10.1371/journal.pone.0353282.r001

**Transfer Alert**

This paper was transferred from another journal. As a result, its full editorial history (including decision letters, peer reviews and author responses) may not be present.

20 Nov 2025

# Peer review: Decision Letter 0

**DOI:** 10.1371/journal.pone.0353282.r002

**Authors:** Edvard Mizsei

29 Jan 2026

PONE-D-25-62362

iNaturalist mammal observations classified by evidence type

PLOS One

Dear Dr. Kays,

Thank you for submitting your manuscript to PLOS ONE. After careful consideration, we feel that it has merit but does not fully meet PLOS ONE’s publication criteria as it currently stands. Therefore, we invite you to submit a revised version of the manuscript that addresses the points raised during the review process.

Both reviewers provided a positive overall evaluation and found the study to be well written and potentially valuable. However, they also raised several points that require revision before the manuscript can be considered further.

In particular, please expand and strengthen the Discussion, which is currently too short. We recommend structuring it to include: (1) interpretation of the results in relation to the stated aims, (2) comparison with existing literature, including how the study fills knowledge gaps or provides methodological advances, (3) limitations of the study, including transferability of results and suggested future research directions, and (4) a brief concluding paragraph summarizing the main findings.

Relatedly, please revise the aims at the end of the Introduction so that they are more explicitly framed around aims, questions, or hypotheses. The Results section and the opening paragraph of the Discussion should clearly follow this revised structure.

Please also ensure that all comments from Reviewer 2 in the attached PDF are explicitly addressed including.

Minor textual and terminological edits suggested by Reviewer 1 should also be implemented, and additional references added where statements currently lack support.

Please submit your revised manuscript by Mar 15 2026 11:59PM. If you will need more time than this to complete your revisions, please reply to this message or contact the journal office at plosone@plos.org. When you're ready to submit your revision, log on to https://www.editorialmanager.com/pone/ and select the 'Submissions Needing Revision' folder to locate your manuscript file.

Please include the following items when submitting your revised manuscript:

- A letter that responds to each point raised by the academic editor and reviewer(s). You should upload this letter as a separate file labeled 'Response to Reviewers'.
- A marked-up copy of your manuscript that highlights changes made to the original version. You should upload this as a separate file labeled 'Revised Manuscript with Track Changes'.
- An unmarked version of your revised paper without tracked changes. You should upload this as a separate file labeled 'Manuscript'.

If you would like to make changes to your financial disclosure, please include your updated statement in your cover letter. Guidelines for resubmitting your figure files are available below the reviewer comments at the end of this letter.

If applicable, we recommend that you deposit your laboratory protocols in protocols.io to enhance the reproducibility of your results. Protocols.io assigns your protocol its own identifier (DOI) so that it can be cited independently in the future. For instructions see: https://journals.plos.org/plosone/s/submission-guidelines#loc-laboratory-protocols. Additionally, PLOS ONE offers an option for publishing peer-reviewed Lab Protocol articles, which describe protocols hosted on protocols.io. Read more information on sharing protocols at https://plos.org/protocols?utm_medium=editorial-email&utm_source=authorletters&utm_campaign=protocols.

We look forward to receiving your revised manuscript.

Kind regards,

Edvard Mizsei

Academic Editor

PLOS One

Journal Requirements:

When submitting your revision, we need you to address these additional requirements.

1. Please ensure that your manuscript meets PLOS ONE's style requirements, including those for file naming. The PLOS ONE style templates can be found at

https://journals.plos.org/plosone/s/file?id=wjVg/PLOSOne_formatting_sample_main_body.pdf and https://journals.plos.org/plosone/s/file?id=ba62/PLOSOne_formatting_sample_title_authors_affiliations.pdf

2. In your Methods section, please include additional information about your dataset and ensure that you have included a statement specifying whether the collection and analysis method complied with the terms and conditions for the source of the data.

3. Thank you for stating the following financial disclosure:

[National Science Foundation Grants 2206783 and 2206784].

Please state what role the funders took in the study.  If the funders had no role, please state: "The funders had no role in study design, data collection and analysis, decision to publish, or preparation of the manuscript."

If this statement is not correct you must amend it as needed.

Please include this amended Role of Funder statement in your cover letter; we will change the online submission form on your behalf.

4. Thank you for stating the following in the Acknowledgments Section of your manuscript:

[Thanks to Michael V. Cove for providing example photos used in Figure 1. Thanks to the North Carolina Museum of Natural Sciences Biodiversity Lab for resources and support. Funding was provided by NSF Awards 2206783 and 2206784.]

We note that you have provided funding information that is currently declared in your Funding Statement. However, funding information should not appear in the Acknowledgments section or other areas of your manuscript. We will only publish funding information present in the Funding Statement section of the online submission form.

Please remove any funding-related text from the manuscript and let us know how you would like to update your Funding Statement. Currently, your Funding Statement reads as follows:

[National Science Foundation Grants 2206783 and 2206784]

Please include your amended statements within your cover letter; we will change the online submission form on your behalf.

5. Please note that your Data Availability Statement is currently missing the repository name and/or the DOI/accession number of each dataset OR a direct link to access each database. If your manuscript is accepted for publication, you will be asked to provide these details on a very short timeline. We therefore suggest that you provide this information now, though we will not hold up the peer review process if you are unable.

6. When completing the data availability statement of the submission form, you indicated that you will make your data available on acceptance. We strongly recommend all authors decide on a data sharing plan before acceptance, as the process can be lengthy and hold up publication timelines. Please note that, though access restrictions are acceptable now, your entire data will need to be made freely accessible if your manuscript is accepted for publication. This policy applies to all data except where public deposition would breach compliance with the protocol approved by your research ethics board. If you are unable to adhere to our open data policy, please kindly revise your statement to explain your reasoning and we will seek the editor's input on an exemption. Please be assured that, once you have provided your new statement, the assessment of your exemption will not hold up the peer review process.

7. We note that Figure 1 in your submission contains images which may be copyrighted. All PLOS content is published under the Creative Commons Attribution License (CC BY 4.0), which means that the manuscript, images, and Supporting Information files will be freely available online, and any third party is permitted to access, download, copy, distribute, and use these materials in any way, even commercially, with proper attribution. For more information, see our copyright guidelines: http://journals.plos.org/plosone/s/licenses-and-copyright.

We require you to either (1) present written permission from the copyright holder to publish these figures specifically under the CC BY 4.0 license, or (2) remove the figures from your submission:

1. You may seek permission from the original copyright holder of Figure 1 to publish the content specifically under the CC BY 4.0 license.

We recommend that you contact the original copyright holder with the Content Permission Form (http://journals.plos.org/plosone/s/file?id=7c09/content-permission-form.pdf) and the following text:

“I request permission for the open-access journal PLOS ONE to publish XXX under the Creative Commons Attribution License (CCAL) CC BY 4.0 (http://creativecommons.org/licenses/by/4.0/). Please be aware that this license allows unrestricted use and distribution, even commercially, by third parties. Please reply and provide explicit written permission to publish XXX under a CC BY license and complete the attached form.”

Please upload the completed Content Permission Form or other proof of granted permissions as an "Other" file with your submission.

In the figure caption of the copyrighted figure, please include the following text: “Reprinted from [ref] under a CC BY license, with permission from [name of publisher], original copyright [original copyright year].”

2. If you are unable to obtain permission from the original copyright holder to publish these figures under the CC BY 4.0 license or if the copyright holder’s requirements are incompatible with the CC BY 4.0 license, please either i) remove the figure or ii) supply a replacement figure that complies with the CC BY 4.0 license. Please check copyright information on all replacement figures and update the figure caption with source information. If applicable, please specify in the figure caption text when a figure is similar but not identical to the original image and is therefore for illustrative purposes only.

8. Please ensure that you refer to Figure 2a in your text as, if accepted, production will need this reference to link the reader to the figure.

If the reviewer comments include a recommendation to cite specific previously published works, please review and evaluate these publications to determine whether they are relevant and should be cited. There is no requirement to cite these works unless the editor has indicated otherwise.

[Note: HTML markup is below. Please do not edit.]

Reviewers' comments:

Reviewer's Responses to Questions

**Comments to the Author**

1. Is the manuscript technically sound, and do the data support the conclusions?

The manuscript must describe a technically sound piece of scientific research with data that supports the conclusions. Experiments must have been conducted rigorously, with appropriate controls, replication, and sample sizes. The conclusions must be drawn appropriately based on the data presented.

Reviewer #1: Yes

Reviewer #2: Yes

**********

2. Has the statistical analysis been performed appropriately and rigorously?

Reviewer #1: Yes

Reviewer #2: I Don't Know

**********

3. Have the authors made all data underlying the findings in their manuscript fully available?

The PLOS Data policy requires authors to make all data underlying the findings described in their manuscript fully available without restriction, with rare exception (please refer to the Data Availability Statement in the manuscript PDF file). The data should be provided as part of the manuscript or its supporting information, or deposited to a public repository. For example, in addition to summary statistics, the data points behind means, medians and variance measures should be available. If there are restrictions on publicly sharing data—e.g. participant privacy or use of data from a third party—those must be specified.

Reviewer #1: Yes

Reviewer #2: Yes

**********

4. Is the manuscript presented in an intelligible fashion and written in standard English?

PLOS ONE does not copyedit accepted manuscripts, so the language in submitted articles must be clear, correct, and unambiguous. Any typographical or grammatical errors should be corrected at revision, so please note any specific errors here.

Reviewer #1: Yes

Reviewer #2: Yes

**********

5. Review Comments to the Author

Please use the space provided to explain your answers to the questions above. You may also include additional comments for the author, including concerns about dual publication, research ethics, or publication ethics. (Please upload your review as an attachment if it exceeds 20,000 characters)

Reviewer #1: The authors describe a machine learning approach that helps categorise iNaturalist mammal observations based on evidence of presence, that could aid further studies involving data collected from the citizen science platform. The paper is well-written, linguistic errors are scarce, if any. I generally liked the topic, although, I feel like that some statements warrant more references. I also think that the discussion is a bit too short, the authors could reflect more on the detected patterns. I think the manuscript could be accepted after addressing a few points.

Line 20: I would change the phrasing ‘participatory scientists’ to citizen science.

Line 22: Change North American to North America

Lines 47 onwards: You should add some references for the expected patterns.

Line 116: I would again change the phrasing of participatory scientists to data collectors or observers

Lines 127 onwards: Do you have any suggestions on how to improve accuracy of the identification of handheld animals? The mentioned groups and I imagine that a significant portion of rodents, shrews and bats that are uploaded as live animals are held in hands.

Lines 162-167: Weren’t any other burrows included in this dataset, for example those of groundhogs or other rodents?

Reviewer #2: An interesting, straightforward manuscript that I enjoyed reading, and that I think will provide a useful resource for future work. I have no major criticisms; see my minor comments/corrections in the attached PDF.

A few other brief comments:

1. Possibly worth stating somewhere (methods? Unsure what best section would be) just for completeness that iNat allows more annotations for any given mammal observation - both for the category 'evidence of presence', and other categories, eg 'alive or dead' - than what you have mentioned/used. For example, iNat users can annotate mammal records as 'hair' or 'construction' (I understand these are lumped into 'other' under your framework, but it would be nice to acknowledge that these options exist/are not lumped within iNat itself), or 'cannot be determined' is an option under dead/alive in iNat. It's not a pressing necessity, but I think it's nice to have that info in there, esp. for future work that cites your paper as an example for annotations.

2. Just noting that I have not commented the technical/methodological elements of the machine learning in your paper (namely lines 193-215), as this is outside my area of expertise.

3. Would be valuable to briefly include a few lines in the discussion explaining/hypothesising why certain mammal orders have observations that are so heavily skewed towards one annotation category, eg the dominance of observations of dead animals for moles + shrews. I know the ecological side of the data isn't the focus of the paper, but it would be interesting information to include for readers and help to provide some context.

**********

6. PLOS authors have the option to publish the peer review history of their article (what does this mean?). If published, this will include your full peer review and any attached files.

If you choose “no”, your identity will remain anonymous but your review may still be made public.

**Do you want your identity to be public for this peer review?**  For information about this choice, including consent withdrawal, please see our Privacy Policy.

Reviewer #1: **Yes:** Bálint Wenner

Reviewer #2: No

**********

[NOTE: If reviewer comments were submitted as an attachment file, they will be attached to this email and accessible via the submission site. Please log into your account, locate the manuscript record, and check for the action link "View Attachments". If this link does not appear, there are no attachment files.]

To ensure your figures meet our technical requirements, please review our figure guidelines: https://journals.plos.org/plosone/s/figures

You may also use PLOS’s free figure tool, NAAS, to help you prepare publication quality figures: https://journals.plos.org/plosone/s/figures#loc-tools-for-figure-preparation.

NAAS will assess whether your figures meet our technical requirements by comparing each figure against our figure specifications.

Attachment Submitted filename: PONE-D-25-62362_reviewer_comments.pdf

# Author comment: Author response to Decision Letter 1

**DOI:** 10.1371/journal.pone.0353282.r003

24 Mar 2026

Dear Dr. Kays,

Thank you for submitting your manuscript to PLOS ONE. After careful consideration, we feel

that it has merit but does not fully meet PLOS ONE’s publication criteria as it currently stands.

Therefore, we invite you to submit a revised version of the manuscript that addresses the points

raised during the review process.

Both reviewers provided a positive overall evaluation and found the study to be well written and

potentially valuable. However, they also raised several points that require revision before the

manuscript can be considered further.

In particular, please expand and strengthen the Discussion, which is currently too short. We

recommend structuring it to include: (1) interpretation of the results in relation to the stated aims,

(2) comparison with existing literature, including how the study fills knowledge gaps or provides

methodological advances, (3) limitations of the study, including transferability of results and

suggested future research directions, and (4) a brief concluding paragraph summarizing the

main findings.

RESPONSE: We have substantially expanded the discussion in response to reviewer

comments. In particular, we added a paragraph that highlights several orders with

larger-than-average proportions of certain evidence types, including hypotheses for these

patterns. We also added a concluding paragraph that places our dataset in context with the

existing annotation system, including some limitations of our data.

Relatedly, please revise the aims at the end of the Introduction so that they are more explicitly

framed around aims, questions, or hypotheses. The Results section and the opening paragraph

of the Discussion should clearly follow this revised structure.

RESPONSE: We have addressed the edits by Reviewer 2 in the attached PDF (see comments

below).

Please also ensure that all comments from Reviewer 2 in the attached PDF are explicitly

addressed including.

RESPONSE: We have addressed the edits by Reviewer 2 in the attached PDF (see comments

below).

Minor textual and terminological edits suggested by Reviewer 1 should also be implemented,

and additional references added where statements currently lack support.

RESPONSE: We have addressed the edits by Reviewer 1 including adding supporting citations

(see comments below).Please submit your revised manuscript by Mar 15 2026 11:59PM. If you will need more time

than this to complete your revisions, please reply to this message or contact the journal office at

plosone@plos.org. When you're ready to submit your revision, log on to

https://www.editorialmanager.com/pone/ and select the 'Submissions Needing Revision' folder to

locate your manuscript file.

Please include the following items when submitting your revised manuscript:

● A letter that responds to each point raised by the academic editor and reviewer(s). You

should upload this letter as a separate file labeled 'Response to Reviewers'.

● A marked-up copy of your manuscript that highlights changes made to the original

version. You should upload this as a separate file labeled 'Revised Manuscript with

Track Changes'.

● An unmarked version of your revised paper without tracked changes. You should

upload this as a separate file labeled 'Manuscript'.

If you would like to make changes to your financial disclosure, please include your updated

statement in your cover letter. Guidelines for resubmitting your figure files are available below

the reviewer comments at the end of this letter.

If applicable, we recommend that you deposit your laboratory protocols in protocols.io to

enhance the reproducibility of your results. Protocols.io assigns your protocol its own identifier

(DOI) so that it can be cited independently in the future. For instructions see:

https://journals.plos.org/plosone/s/submission-guidelines#loc-laboratory-protocols. Additionally,

PLOS ONE offers an option for publishing peer-reviewed Lab Protocol articles, which describe

protocols hosted on protocols.io. Read more information on sharing protocols at

https://plos.org/protocols?utm_medium=editorial-email&utm_source=authorletters&utm_campai

gn=protocols.

We look forward to receiving your revised manuscript.

Kind regards,

Edvard Mizsei

Academic Editor

PLOS OneReviewer #1: The authors describe a machine learning approach that helps categorise

iNaturalist mammal observations based on evidence of presence, that could aid further studies

involving data collected from the citizen science platform. The paper is well-written, linguistic

errors are scarce, if any. I generally liked the topic, although, I feel like that some statements

warrant more references. I also think that the discussion is a bit too short, the authors could

reflect more on the detected patterns. I think the manuscript could be accepted after addressing

a few points.

RESPONSE: Thank you for the positive feedback and for taking the time to review this

manuscript. In response to this review, we have included more references to support

statements. We have also expanded on the discussion to reflect more on patterns presented.

Line 20: I would change the phrasing ‘participatory scientists’ to citizen science.

RESPONSE: Changed as requested.

Line 22: Change North American to North America

RESPONSE: Thank you, we’ve fixed this typo.

Lines 47 onwards: You should add some references for the expected patterns.

RESPONSE: We’ve added references here, thanks for the suggestion.

Line 116: I would again change the phrasing of participatory scientists to data collectors or

observers

RESPONSE: Changed.

Lines 127 onwards: Do you have any suggestions on how to improve accuracy of the

identification of handheld animals? The mentioned groups and I imagine that a significant

portion of rodents, shrews and bats that are uploaded as live animals are held in hands.

RESPONSE: We have added text to the Discussion describing what we think it would take to

change these.Lines 162-167: Weren’t any other burrows included in this dataset, for example those of

groundhogs or other rodents?

RESPONSE: Thanks for highlighting this point of confusion. We have rewritten this section for

clarity. As the reviewer notes, a wide and representative variety of species were included in the

training data. We specifically augmented the data with extra observations from select, common

species with lots of sign observations to try to increase the total number of sign observations as

much as we could.

Reviewer #2: An interesting, straightforward manuscript that I enjoyed reading, and that I think

will provide a useful resource for future work. I have no major criticisms; see my minor

comments/corrections in the attached PDF.

RESPONSE: Thanks for the positive feedback and for your time spent reviewing this

manuscript.

A few other brief comments:

1. Possibly worth stating somewhere (methods? Unsure what best section would be) just for

completeness that iNat allows more annotations for any given mammal observation - both for

the category 'evidence of presence', and other categories, eg 'alive or dead' - than what you

have mentioned/used. For example, iNat users can annotate mammal records as 'hair' or

'construction' (I understand these are lumped into 'other' under your framework, but it would be

nice to acknowledge that these options exist/are not lumped within iNat itself), or 'cannot be

determined' is an option under dead/alive in iNat. It's not a pressing necessity, but I think it's

nice to have that info in there, esp. for future work that cites your paper as an example for

annotations.

RESPONSE: We have added text to the discussion that explains that our model is intended to

supplement, not replace, the iNaturalist annotation system, and now include a discussion of the

benefits and drawbacks of our dataset versus the integrated iNaturalist annotations.

2. Just noting that I have not commented the technical/methodological elements of the machine

learning in your paper (namely lines 193-215), as this is outside my area of expertise.

RESPONSE: Thanks for noting this.3. Would be valuable to briefly include a few lines in the discussion explaining/hypothesising

why certain mammal orders have observations that are so heavily skewed towards one

annotation category, eg the dominance of observations of dead animals for moles + shrews. I

know the ecological side of the data isn't the focus of the paper, but it would be interesting

information to include for readers and help to provide some context.

RESPONSE: We have added such a paragraph to the discussion, describing patterns in

evidence type variation by order and offering some hypotheses as to the ecological processes

behind these patterns.

[Comments in annotated PDF]

RESPONSE: We corrected the manuscript to resolve a number of points where the text or

clarity of the manuscript needed improving. Small changes made in response to R2 included:

● Capitalized “Research Grade” throughout the manuscript and clarified that Research

Grade observations must meet quality standards above and beyond the inclusion of

evidence

● Clarifying a number of points where the first draft was vague (such as the phrase “when

necessary”)

● Made a number of corrections for methodological completeness, providing more

complete rationale for ML methodology including

○ Why we excluded low-quality records (they reduce overall model performance)

○ What it would take to classify bats

○ How annotators decided when to go to the iNaturalist website for additional

context

● Modified figures and legends as requested

● Substantially expanded the Discussion section to describe order-by-order patterns in

classified data

● Included new discussion about context and visual cues used by human observers

# Peer review: Decision Letter 1

**DOI:** 10.1371/journal.pone.0353282.r004

**Authors:** Edvard Mizsei, Edvard Mizsei

6 Apr 2026

PONE-D-25-62362R1

iNaturalist mammal observations classified by evidence type

PLOS One

Dear Dr. Kays,

Thank you for submitting your manuscript to PLOS ONE. After careful consideration, we feel that it has merit but does not fully meet PLOS ONE’s publication criteria as it currently stands. Therefore, we invite you to submit a revised version of the manuscript that addresses the points raised during the review process.

Please address the "very small handful of super minor comments" of reviewer 2 before the manuscript can be accepted. Otherwise, the reviewers were satisfied the revision.

Please submit your revised manuscript by May 21 2026 11:59PM. If you will need more time than this to complete your revisions, please reply to this message or contact the journal office at plosone@plos.org. When you're ready to submit your revision, log on to https://www.editorialmanager.com/pone/ and select the 'Submissions Needing Revision' folder to locate your manuscript file.

Please include the following items when submitting your revised manuscript:

- A letter that responds to each point raised by the academic editor and reviewer(s). You should upload this letter as a separate file labeled 'Response to Reviewers'.
- A marked-up copy of your manuscript that highlights changes made to the original version. You should upload this as a separate file labeled 'Revised Manuscript with Track Changes'.
- An unmarked version of your revised paper without tracked changes. You should upload this as a separate file labeled 'Manuscript'.

If you would like to make changes to your financial disclosure, please include your updated statement in your cover letter. Guidelines for resubmitting your figure files are available below the reviewer comments at the end of this letter.

If applicable, we recommend that you deposit your laboratory protocols in protocols.io to enhance the reproducibility of your results. Protocols.io assigns your protocol its own identifier (DOI) so that it can be cited independently in the future. For instructions see: https://journals.plos.org/plosone/s/submission-guidelines#loc-laboratory-protocols. Additionally, PLOS ONE offers an option for publishing peer-reviewed Lab Protocol articles, which describe protocols hosted on protocols.io. Read more information on sharing protocols at https://plos.org/protocols?utm_medium=editorial-email&utm_source=authorletters&utm_campaign=protocols.

As the corresponding author, your ORCID iD is verified in the submission system and will appear in the published article. PLOS supports the use of ORCID, and we encourage all coauthors to register for an ORCID iD and use it as well. Please encourage your coauthors to verify their ORCID iD within the submission system before final acceptance, as unverified ORCID iDs will not appear in the published article. *Only*  the individual author can complete the verification step; PLOS staff *cannot*  verify ORCID iDs on behalf of authors.

We look forward to receiving your revised manuscript.

Kind regards,

Edvard Mizsei

Academic Editor

PLOS One

Journal Requirements:

1. If the reviewer comments include a recommendation to cite specific previously published works, please review and evaluate these publications to determine whether they are relevant and should be cited. There is no requirement to cite these works unless the editor has indicated otherwise.

2. Please review your reference list to ensure that it is complete and correct. If you have cited papers that have been retracted, please include the rationale for doing so in the manuscript text, or remove these references and replace them with relevant current references. Any changes to the reference list should be mentioned in the rebuttal letter that accompanies your revised manuscript. If you need to cite a retracted article, indicate the article’s retracted status in the References list and also include a citation and full reference for the retraction notice.

[Note: HTML markup is below. Please do not edit.]

Reviewers' comments:

Reviewer's Responses to Questions

**Comments to the Author**

1. If the authors have adequately addressed your comments raised in a previous round of review and you feel that this manuscript is now acceptable for publication, you may indicate that here to bypass the “Comments to the Author” section, enter your conflict of interest statement in the “Confidential to Editor” section, and submit your "Accept" recommendation.

Reviewer #1: All comments have been addressed

Reviewer #2: (No Response)

**********

2. Is the manuscript technically sound, and do the data support the conclusions?

The manuscript must describe a technically sound piece of scientific research with data that supports the conclusions. Experiments must have been conducted rigorously, with appropriate controls, replication, and sample sizes. The conclusions must be drawn appropriately based on the data presented.

Reviewer #1: Yes

Reviewer #2: Yes

**********

3. Has the statistical analysis been performed appropriately and rigorously?

Reviewer #1: Yes

Reviewer #2: Yes

**********

4. Have the authors made all data underlying the findings in their manuscript fully available?

The PLOS Data policy requires authors to make all data underlying the findings described in their manuscript fully available without restriction, with rare exception (please refer to the Data Availability Statement in the manuscript PDF file). The data should be provided as part of the manuscript or its supporting information, or deposited to a public repository. For example, in addition to summary statistics, the data points behind means, medians and variance measures should be available. If there are restrictions on publicly sharing data—e.g. participant privacy or use of data from a third party—those must be specified.

Reviewer #1: Yes

Reviewer #2: Yes

**********

5. Is the manuscript presented in an intelligible fashion and written in standard English?

PLOS ONE does not copyedit accepted manuscripts, so the language in submitted articles must be clear, correct, and unambiguous. Any typographical or grammatical errors should be corrected at revision, so please note any specific errors here.

Reviewer #1: Yes

Reviewer #2: Yes

**********

6. Review Comments to the Author

Please use the space provided to explain your answers to the questions above. You may also include additional comments for the author, including concerns about dual publication, research ethics, or publication ethics. (Please upload your review as an attachment if it exceeds 20,000 characters)

Reviewer #1: The authors have adressed my previous comments, therefore I can suggest acceptance. I found the revised manuscript appropriate for publication.

Reviewer #2: Thanks for the thorough revision; I've reattached the PDF with a very small handful of super minor comments, other than those everything looks great (please note that I left my comments on the tracked changes version of the manuscript in the PDF)

**********

7. PLOS authors have the option to publish the peer review history of their article (what does this mean?). If published, this will include your full peer review and any attached files.

If you choose “no”, your identity will remain anonymous but your review may still be made public.

**Do you want your identity to be public for this peer review?**  For information about this choice, including consent withdrawal, please see our Privacy Policy.

Reviewer #1: **Yes:** Bálint Wenner

Reviewer #2: No

**********

[NOTE: If reviewer comments were submitted as an attachment file, they will be attached to this email and accessible via the submission site. Please log into your account, locate the manuscript record, and check for the action link "View Attachments". If this link does not appear, there are no attachment files.]

To ensure your figures meet our technical requirements, please review our figure guidelines: https://journals.plos.org/plosone/s/figures

You may also use PLOS’s free figure tool, NAAS, to help you prepare publication quality figures: https://journals.plos.org/plosone/s/figures#loc-tools-for-figure-preparation.

NAAS will assess whether your figures meet our technical requirements by comparing each figure against our figure specifications.

Attachment Submitted filename: PONE-D-25-62362_reviewer_comments.pdf

# Author comment: Author response to Decision Letter 2

**DOI:** 10.1371/journal.pone.0353282.r005

5 Jun 2026

We received no reviewer comments from the editors in the last round of review so we have made no changes.

# Editor report: Decision Letter 2

**DOI:** 10.1371/journal.pone.0353282.r006

**Authors:** Edvard Mizsei, Edvard Mizsei, Edvard Mizsei

22 Jun 2026

iNaturalist mammal observations classified by evidence type

PONE-D-25-62362R2

Dear Dr. Kays,

We’re pleased to inform you that your manuscript has been judged scientifically suitable for publication and will be formally accepted for publication once it meets all outstanding technical requirements.

Within one week, you’ll receive an e-mail detailing the required amendments. When these have been addressed, you’ll receive a formal acceptance letter and your manuscript will be scheduled for publication.

An invoice will be generated when your article is formally accepted. Please note, if your institution has a publishing partnership with PLOS and your article meets the relevant criteria, all or part of your publication costs will be covered. Please make sure your user information is up-to-date by logging into Editorial Manager at Editorial Manager® and clicking the ‘Update My Information' link at the top of the page. For questions related to billing, please contact billing support.

If your institution or institutions have a press office, please notify them about your upcoming paper to help maximize its impact. If they’ll be preparing press materials, please inform our press team as soon as possible -- no later than 48 hours after receiving the formal acceptance. Your manuscript will remain under strict press embargo until 2 pm Eastern Time on the date of publication. For more information, please contact onepress@plos.org.

Kind regards,

Edvard Mizsei

Academic Editor

PLOS One

Additional Editor Comments (optional):

Reviewers' comments:

# Editor report: Acceptance letter

**DOI:** 10.1371/journal.pone.0353282.r007

**Authors:** Edvard Mizsei, Edvard Mizsei, Edvard Mizsei

PONE-D-25-62362R2

PLOS One

Dear Dr. Kays,

I'm pleased to inform you that your manuscript has been deemed suitable for publication in PLOS One. Congratulations! Your manuscript is now being handed over to our production team.

At this stage, our production department will prepare your paper for publication. This includes ensuring the following:

* All references, tables, and figures are properly cited

* All relevant supporting information is included in the manuscript submission,

* There are no issues that prevent the paper from being properly typeset

You will receive further instructions from the production team, including instructions on how to review your proof when it is ready. Please keep in mind that we are working through a large volume of accepted articles, so please give us a few days to review your paper and let you know the next and final steps.

Lastly, if your institution or institutions have a press office, please let them know about your upcoming paper now to help maximize its impact. If they'll be preparing press materials, please inform our press team within the next 48 hours. Your manuscript will remain under strict press embargo until 2 pm Eastern Time on the date of publication. For more information, please contact onepress@plos.org.

You will receive an invoice from PLOS for your publication fee after your manuscript has reached the completed accept phase. If you receive an email requesting payment before acceptance or for any other service, this may be a phishing scheme. Learn how to identify phishing emails and protect your accounts at https://explore.plos.org/phishing.

If we can help with anything else, please email us at customercare@plos.org.

Thank you for submitting your work to PLOS ONE and supporting open access.

Kind regards,

PLOS ONE Editorial Office Staff

on behalf of

Dr. Edvard Mizsei

Academic Editor

PLOS One
